# Self-Care Practices Among Adults With Type 2 Diabetes Mellitus in Urban and Rural Areas of Bhubaneswar, India

**DOI:** 10.7759/cureus.117580

**Published:** 2026-10-07

**Authors:** Smriti Madhusikta, Smrutiranjan Nayak, Alpana Mishra

**Affiliations:** 1 Community Medicine, Institute of Medical Sciences (IMS) and SUM Hospital, Bhubaneswar, IND; 2 Community Medicine, Kalinga Institute of Medical Sciences, Bhubaneswar, IND

**Keywords:** chronic disease, diabetes mellitis, lifestyle diseases, non-communicable disease care, sdsca, self-care, type 2 diabetes mellitus, urban–rural comparison

## Abstract

Background: Type 2 diabetes mellitus (T2DM) is a rapidly growing public health problem, particularly in low- and middle-income countries. Effective self-care practices are essential for optimal glycemic control and prevention of complications. Urbanization has introduced lifestyle and health system-related challenges that may differentially influence diabetes self-care behaviors.

Objectives: The objective of the study is to assess self-care practices among adults with T2DM in Bhubaneswar and to compare these practices between urban and rural populations.

Methods: A community-based study was conducted from December 2020 to September 2022 among 465 adults with T2DM using multistage cluster sampling. The study design was cross-sectional. Self-care practices were assessed using the Summary of Diabetes Self-Care Activities (SDSCA) scale. Data were analyzed using SPSS version 22.0 (IBM Corp., Armonk, NY, USA). Chi-squared test, Fisher’s exact test, and crude odds ratio were reported.

Results: Poor dietary habits (p = 0.035) and exercise behaviors (p = 0.018) were significantly more prevalent among urban participants, while rural participants demonstrated better blood glucose monitoring (p = 0.001). Residence showed a significant association with multiple dimensions of self-care. Women had significantly less smoking habit (p = 0.005). Poor dietary habits (p = 0.001) were significantly more prevalent among participants with uncontrolled blood sugar, whereas poor exercise, although more prevalent, was not statistically significant (p = 0.080) in the same group.

Conclusion: Urban residents exhibited poorer lifestyle-related self-care behaviors despite better healthcare access, highlighting the need for targeted urban diabetes interventions. Poor dietary and exercise habits are common lapses in self-care behavior and should be more emphasized.

## Introduction

Diabetes mellitus is one of the most important non-communicable diseases worldwide. According to the IDF Diabetes Atlas (2021), an estimated 537 million adults aged 20-79 years were living with diabetes, and this number is projected to increase to 643 million by 2030 and 783 million by 2045. The prevalence has risen more rapidly in low- and middle-income countries, including India. In 2019, approximately 1.5 million deaths were directly attributed to diabetes, with 48% of these deaths occurring among individuals younger than 70 years of age [1].

Optimal glycemic control remains the cornerstone of diabetes management, as it prevents or delays the development of microvascular and macrovascular complications, thereby reducing morbidity, mortality, and healthcare costs [2]. Self-care is defined as the process of managing illness and maintaining health through various health-promoting practices. It is a fundamental component of diabetes management and encompasses a range of behaviors, including healthy eating, regular physical activity, adherence to prescribed medication, blood glucose monitoring, foot care, and avoidance of tobacco use [3-5]. Sustained adherence to these self-care practices has been shown to improve glycemic control, reduce diabetes-related complications, and enhance quality of life [6].

Urbanization has been associated with substantial lifestyle changes, including sedentary occupations, reduced physical activity, increased consumption of energy-dense processed foods, psychosocial stress, irregular meal patterns, and a rising prevalence of obesity [7,8]. Despite having better physical access to healthcare facilities, urban residents often demonstrate suboptimal self-care practices because of time constraints, work-related stress, inadequate counseling, and greater reliance on pharmacological treatment [9,10]. Furthermore, higher out-of-pocket healthcare expenditure in urban settings may adversely affect long-term diabetes management [11].

Urban-rural differences in lifestyle, occupational activity, dietary patterns, healthcare utilization, and social support systems can significantly influence diabetes self-care practices. Rural populations often engage in higher levels of physical activity through agricultural work, manual labor, and household activities, whereas urban populations are more likely to lead sedentary lifestyles [12-14].

Given the marked differences in living conditions, lifestyle behaviors, and healthcare access between urban and rural populations, understanding the context-specific barriers and facilitators of diabetes self-care is essential for developing effective, location-specific interventions. However, evidence comparing diabetes self-care practices among urban and rural populations in eastern India remains limited. Therefore, the present study was undertaken to assess self-care practices among adults with type 2 diabetes mellitus (T2DM) residing in urban and rural areas of Bhubaneswar, Odisha. Further comparison of self-care activities with important sociodemographic factors was also conducted.

## Materials and methods

This cross-sectional study was conducted in Bhubaneswar from December 2020 to September 2022. Adults aged 18-70 years, residing in the study area for at least one year, diagnosed with T2DM for a minimum duration of six months, and receiving treatment were included. Pregnant women and individuals with chronic debilitating illnesses were excluded.

Bhubaneswar, the capital of Odisha, has experienced rapid urbanization over the past two decades, accompanied by significant changes in dietary habits, lifestyle, and utilization of healthcare. The surrounding rural areas have different socioeconomic characteristics, healthcare access, and health-seeking behaviors. These contrasting urban and rural settings within the same geographical region provide an appropriate context for examining differences in diabetes self-care practices and identifying population-specific barriers that can inform targeted public health interventions.

The study design was cross-sectional. The minimum sample size required for a cross-sectional study was calculated using the following formula: n = [Z2pq/d2] * design effect, where design effect was taken as 1.2. To obtain the highest possible sample size, prevalence was taken as 50%.

*n = (1.96 &times; 1.96 &times; 50 &times; 50)/52 &times; 1.2 = (9,604/25) &times; 1.2= 384.16 &times; 1.2*

*n= 460.99 &asymp; 461* (minimum sample size required)

A multistage random sampling technique was employed. Urban areas under Bhubaneswar Municipal Corporation were stratified into three administrative zones having wards: North (21 wards), South West (21 wards), and South East (25 wards). Five wards were randomly selected, and the sample size was equally distributed among the selected wards. Bhubaneswar Block consists of 20 Gram Panchayats (GPs), from which five GPs were selected randomly. All the villages were enumerated, one village was selected from each selected GP by simple random sampling, and the sample size was equally distributed among the study clusters. The total sample size of 461 was distributed between urban and rural areas (Figure 1). The list of adults with T2DM was obtained from Accredited Social Health Activists (ASHAs), and 47 participants were selected randomly. In case the required number was not reached in the same village, eligible participants from adjacent villages were included.

**Figure 1:**
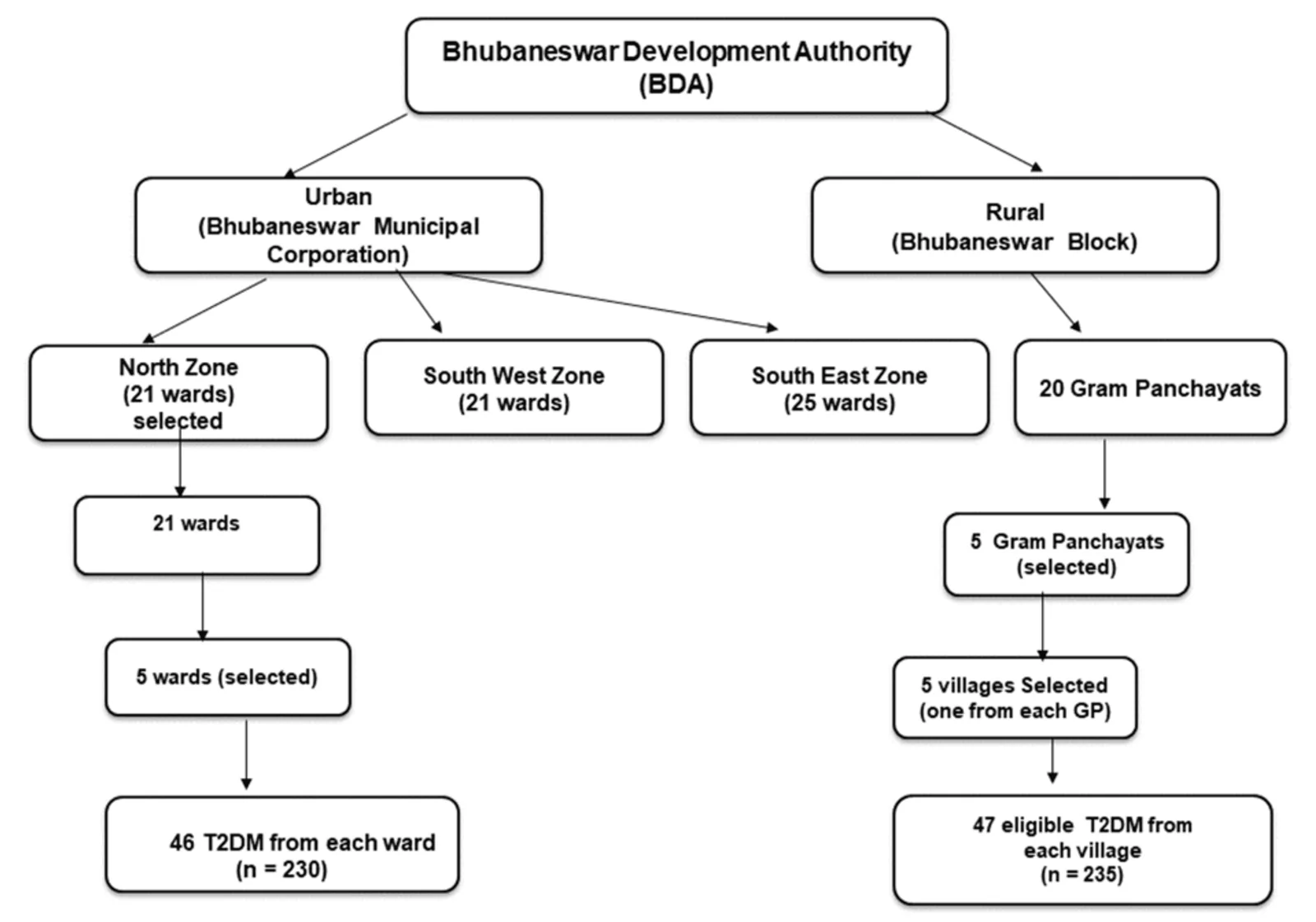
Multistage sampling procedure T2DM: type 2 diabetes mellitus

Data were collected using a semi-structured questionnaire comprising sociodemographic details and the revised Summary of Diabetes Self-Care Activities (SDSCA) scale [15]. Ethical approval was obtained from the Institutional Ethics Committee with letter no. KIIT/KIMS/IEC/420/2020, and informed written consent was obtained from all participants.

Statistical analysis was performed using SPSS version 22.0 (IBM Corp., Armonk, NY, USA). Descriptive statistics, chi-squared test, Fisher’s exact test, and unadjusted odds ratio (OR) with 95% confidence intervals (CIs) were used as appropriate.

Variables

WHO’s definition of self-care is the ability of individuals, families, and communities to promote their own health, prevent disease, maintain health, and cope with illness and disability with or without the support of a health worker [16]. Self-care interventions include evidence-based, quality drugs, devices, diagnostics, and/or digital products that can be provided fully or partially outside of formal health services and can be used with or without a health worker [17]. The SDSCA is a brief self-reporting instrument for measuring levels of self-management across different components of the diabetes regimen. The revised SDSCA consists of a core set of 11 items from the original scale along with the expanded list of 14 additional questions. The revised version also includes items on foot care and cigarette smoking. After obtaining scores for the individual items for the revised SDSCA scale, mean scores were obtained for individual domains. These were recoded, as a mean score of >4 was considered good dietary behavior and good foot care practice. A mean score of >5 was taken for good exercise behavior, and not smoking was considered appropriate self-care behavior. For glycemic control assessment, an HbA1c level of ≤7% was considered indicative of good glycemic control, while >7% represented poor control [17].

## Results

A total of 465 participants were included in the study, with a mean age of 50.08 ± 10.63 years. As indicated in Table 1, the majority of study participants (322 (69.2%)) were males, and approximately half (47.5%) were in the age group of 36-50 years. About half (51.4%) of the participants resided in rural areas, and the majority (89.2%) were Hindus. Based on the B.G. Prasad socioeconomic classification (May 2021), 27.3% belonged to the upper socioeconomic class [18].

**Table 1:** Sociodemographic and clinical characteristics of the study participants (N = 465) Values are presented as n (%) for categorical variables and mean ± standard deviation (SD) for continuous variables. BMI: body mass index

Variable	Category	Value
Age (years)	18-35	35 (7.5)
	36-50	221 (47.5)
	51-70	209 (44.9)
Residence	Rural	239 (51.4)
	Urban	226 (48.6)
Religion	Hindu	415 (89.2)
	Muslim	34 (7.3)
	Christian	16 (3.4)
Socioeconomic class	I (upper)	127 (27.3)
	II (upper middle)	116 (24.9)
	III (middle)	55 (11.8)
	IV (lower middle)	81 (17.4)
	V (lower)	86 (18.5)
Type of family	Nuclear	229 (49.2)
	Joint	135 (29.0)
	Three-generation	101 (21.7)
Height (cm)	Mean ± SD	167.17 ± 9.47
Weight (kg)	Mean ± SD	72.52 ± 15.90
BMI (kg/m²)	Mean ± SD	25.88 ± 4.63
Fasting blood sugar (mg/dL) (n = 403)	Mean ± SD	156.42 ± 29.63
Postprandial blood sugar (mg/dL) (n = 445)	Mean ± SD	223.27 ± 44.84

The mean body mass index (BMI) of the study participants was 25.88 ± 4.63, which falls into the obese category according to the Asian Indian classification for adult BMI. The mean height of participants was 167.17 ± 9.47 cm, and the mean weight was 72.52 ± 15.90 kg. Men demonstrated a higher mean BMI (26.21 ± 4.64) compared to women (25.10 ± 4.53). The mean fasting blood glucose (FBS) of the study participants (n = 403) was 156.42 ± 29.63 mg/dL, and the mean postprandial blood sugar (PPBS) was 223.27 ± 44.84 mg/dL. The mean HbA1c level was 6.99 ± 0.83 mmol/mol.

Table 2 illustrates the associations between self-care activities and sociodemographic characteristics. The analysis revealed that the majority (52.04%) of the study population did not exhibit good dietary behavior, which was significantly associated with young age, 18 to 50 years (OR 1.562; 95% CI: 1.082-2.257), and residence in rural areas (OR 1.483; 95% CI: 1.029-2.138).

**Table 2:** Association of dietary habit, medication adherence, and exercise with sociodemographic and diabetic parameters (N = 465) *Reference category OR: crude odds ratio; p: p-value; CI: confidence interval

Variables	Poor diet, n (%)	OR (95% CI)	χ²	p	Poor medication, n (%)	OR (95% CI)	χ²	p	Poor exercise, n (%)	OR (95% CI)	χ²	p
Gender												
Male n = 322	170 (52.8)	1.10 (0.74-1.63)	0.237	0.626	77 (23.9)	0.93 (0.59-1.47)	0.086	0.770	225 (69.9)	0.84 (0.54-1.31)	0.606	0.436
Female* n = 143	72 (50.3)	Ref.	-	-	36 (25.2)	Ref.	-	-	105 (73.4)	Ref.	-	-
Age												
18-50 yrs n = 256	146 (57.0)	1.56 (1.08-2.26)	5.678	0.012	73 (28.5)	1.68 (1.08-2.61)	5.500	0.019	170 (66.4)	0.61 (0.40-0.91)	5.752	0.016
>50 yrs* n = 209	96 (45.9)	Ref.	-	-	40 (19.1)	Ref.	-	-	160 (76.6)	Ref.	-	-
Residence												
Urban n = 226	129 (57.1)	1.48 (1.03-2.14)	4.469	0.035	53 (23.5)	0.91 (0.60-1.40)	0.173	0.678	172 (76.1)	1.63 (1.09-2.45)	5.635	0.018
Rural* n = 239	113 (47.3)	Ref.	-	-	60 (25.1)	Ref.	-	-	158 (66.1)	Ref.	-	-
Duration of diabetes												
<5 yrs n = 224	122 (54.5)	1.21 (0.84-1.74)	1.015	0.314	61 (27.2)	1.36 (0.89-2.08)	2.018	0.155	158 (70.5)	0.96 (0.64-1.43)	0.039	0.843
≥5 yrs* n = 241	120 (49.8)	Ref.	-	-	52 (21.6)	Ref.	-	-	172 (71.4)	Ref.	-	-
Blood sugar												
Controlled n = 253	113 (44.7)	0.52 (0.36-0.75)	11.467	0.001	62 (24.5)	1.03 (0.67-1.57)	0.013	0.910	171 (67.6)	0.70 (0.46-1.05)	3.075	0.080
Poorly controlled* n = 212	129 (60.8)	Ref.	-	-	51 (24.1)	Ref.	-	-	159 (75.0)	Ref.	-	-

Table 3 illustrates that good medication adherence (75.7%) was observed across all three independent variables, with statistically significantly higher medication adherence (80.9%) noted among the >50 years age group (OR 1.685; 95% CI: 1.087-2.613). In Table 2, participants aged >50 years demonstrated poorer exercise behavior compared to those in the 18-50 years age group (OR 0.605; 95% CI: 0.401-0.914). Respondents residing in rural areas exhibited significantly better exercise behavior than those in urban areas (OR 1.633; 95% CI: 1.088-2.452).

**Table 3:** Association of blood sugar monitoring, foot care, and smoking habit components of SDSCA with sociodemographic and diabetic parameters (N = 465) *Reference category OR: crude odds ratio; SDSCA: Summary of Diabetes Self-Care Activities; CI: confidence interval

Variables	Poor blood sugar monitoring, n (%)	Crude OR (95% CI)	χ²	p-value	Poor foot care, n (%)	Crude OR (95% CI)	χ²	p-value	Positive smoking history, n (%)	Crude OR (95% CI)	χ²	p-value
Gender												
Male n = 322	95 (29.5)	0.690 (0.456-1.044)	3.102	0.078	223 (69.3)	1.034 (0.676-1.582)	0.024	0.876	17 (5.3)	1.469 (1.379-1.565)	7.836	0.005
Female* n = 143	54 (37.8)	Ref.	-	-	98 (68.5)	Ref.	-	-	0 (0)	Ref.	-	-
Age												
18-50 yrs n = 256	76 (29.7)	0.787 (0.532-1.163)	1.451	0.228	165 (64.5)	0.616 (0.412-0.922)	5.587	0.018	6 (2.3)	0.432 (0.152-1.189)	2.784	0.095
>50 yrs* n = 209	73 (34.9)	Ref.	-	-	156 (74.6)		-	-	11 (5.3)		-	-
Residence												
Urban n = 226	100 (44.2)	3.077 (2.044-4.634)	30.078	<0.001	172 (76.1)	1.924 (1.287-2.877)	10.293	<0.001	4 (1.8)	0.313 (0.101-0.975)	4.440	0.046
Rural* n = 239	49 (20.5)		-	-	149 (62.3)		-	-	13 (5.4)		-	-
Duration of diabetes												
<5 yrs n = 224	60 (26.8)	0.625 (0.421-0.927)	5.486	0.019	146 (65.2)	0.706 (0.476-1.047)	3.002	0.083	7 (3.1)	0.745 (0.279-1.992)	0.346	0.556
≥5 yrs* n = 241	89 (36.9)	Ref.	-	-	175 (72.6)	Ref.	-	-	10 (4.1)	Ref.	-	-
Blood sugar												
Controlled n = 253	84 (33.2)	1.124 (0.760-1.664)	0.342	0.559	172 (68.0)	0.898 (0.604-1.334)	0.285	0.593	6 (2.4)	0.444 (0.161-1.221)	2.599	0.107
Poorly controlled* n = 212	65 (30.7)		-	-	149 (70.3)		-	-	11 (5.2)		-	-

Foot care demonstrated a statistically significant association with residential area (OR 1.924; 95% CI: 1.287-2.877). A statistically significant association was observed between smoking status and gender (OR 1.469; 95% CI: 1.379-1.565).

Among participants with <5 years of diabetes duration, 29.5% demonstrated good exercise behavior. Study participants with >5 years of diabetes duration exhibited poorer blood glucose monitoring behavior than those with diabetes duration < 5 years (OR 0.625; 95% CI: 0.421-0.927).

## Discussion

This study describes the different components of self-care practices of diabetes patients residing in both urban and rural areas of Bhubaneswar, Odisha. Among the various aspects of self-care activities, this study shows that men (30.1%) have better exercise behavior than women (26.6%), which is similar to a study done in an urban slum in Hyderabad [19]. Men have more access to physical activity opportunities compared to women, which explains the behavior. In the present study, people with diabetes in the lower age group (18-50 years) were 1.6 times more likely to have poor dietary practice and 1.7 times more likely to have poor medication adherence as compared to people in the age group > 50 years, which is similar to a study done in Ethiopia that states that age is a factor associated with poor self-care practice [20]. This may be due to the fact that the majority of the younger population is working, and poor dietary and medication compliance may be related to their work lifestyle, whereas the elderly have the opportunity to have home-cooked food with better dietary practices. On the contrary, participants of 18-50 years had better exercise behavior, which may be due to their active involvement in occupation. In the present study, participants residing in urban areas had 1.5 times higher chances of having poor diet, which is similar to a study done in Mangalore, Karnataka [21]. However, poor dietary practice was found to be unsatisfactory in the majority of people residing in rural areas (64.8%) in a study done in Chennai [22]; in the current study, 47.3% of the rural participants had poor dietary practice. This may be due to the difference in geographical area and different dietary patterns of the population. The majority of the urban population (76.1%) in this study had poor exercise behavior, which is similar to a study in Karnataka [21] and can be due to the busy lifestyle and lack of space for exercise in the neighborhood for urban residents.

Participants with controlled glycemic index had lower chances of having a poor diet, which is similar to a study done in Brazil [23], as they are inversely related; better dietary habits lead to better glycemic control in general. This also emphasizes the importance of awareness and motivation to have better glycemic control. Smoking history was found to be statistically significant with sex, as female smoking is culturally not acceptable in Odisha. Along with this, the participants residing in urban areas had about three times more chances of poor blood sugar monitoring behavior and about two times higher chances of poor foot care. In urban areas, healthcare services by primary health care providers are limited in contrast to rural areas. Better service delivery and care provision due to structured healthcare and long-term residence of the population in rural areas may have led to better access to non-communicable disease management services. Rural participants exhibited better physical activity, monitoring behaviors, and foot care, likely reflecting lifestyle-related physical exertion, cleaning of feet after working barefoot, and access to free services under public health programs like the National Programme for Prevention and Control of Non-Communicable Diseases (NPNCD) [22,23]. Despite better healthcare availability, urban residents exhibited poorer self-care, emphasizing the need for structured lifestyle counseling, workplace-based interventions, and strengthened primary care follow-up in urban settings.

The cross-sectional design limits causal inference. Self-reported data may be subject to recall and social desirability bias. The COVID-19 pandemic may have influenced self-care behaviors during the study period.

## Conclusions

Self-care practices among adults with T2DM varied significantly between urban and rural populations. Dietary and exercise behaviors were significantly poorer than medication adherence and blood sugar monitoring. Most patients were advised a low-fat, no-sweets diet and at least 30 minutes of daily physical activity. Rural participants showed better monitoring and physical activity, likely due to their more active daily lifestyle despite their low engagement in diabetes-specific exercise. However, they received limited counseling beyond medication and monitoring. Advice on foot care and long-term complications was largely lacking, resulting in significantly poor foot care across all variables.

Increased awareness through information, education, and communication (IEC) about various government provisions under the NPNCD program should be promoted, particularly in urban areas where out-of-pocket expenditure is higher. Healthcare providers should emphasize comprehensive dietary advice apart from reduced sweets and carbohydrates, including complex carbohydrates, high-fiber diets, and regular meal timing, along with a stronger focus on foot care and eye care.
